# The Aqueous Extract of *Sclerocarya birrea*, *Nauclea latifolia*, and *Piper longum* Mixture Protects Striatal Neurons and Movement-Associated Functionalities in a Rat Model of Diabetes-Induced Locomotion Dysfunction

**DOI:** 10.1155/2023/7865919

**Published:** 2023-07-04

**Authors:** Jean Philippe Djientcheu Tientcheu, Florence Ngueguim Tsofack, Racéline Kamkumo Gounoue, Rodrigue Ngapout Fifen, Paul Désiré Djomeni Dzeufiet, Théophile Dimo

**Affiliations:** Laboratory of Animal Physiology, Faculty of Science, University of Yaoundé 1, P.O. Box 812, Yaoundé, Cameroon

## Abstract

Among the many complications of type 2 diabetes (T2D), locomotor disorders have been poorly studied and understood. Therefore, no disease-modifying treatment is usually considered. The study aimed to investigate the effect of the aqueous extract of *Sclerocarya birrea*, *Nauclea latifolia*, and *Piper longum* (SNP) mixture on locomotor activity in fructose/streptozotocin-induced diabetic rats. T2D was induced by 10% fructose orally (6 weeks) and streptozotocin (STZ, 35 mg/kg, *i.v.*) in 25 male rats. Diabetic animals received distilled water, metformin (200 mg/kg), or the aqueous extract of the SNP mixture (75, 150, or 300 mg/kg). A 10-minute open field test was performed in diabetic rats (glycemia: 126 and 350 mg/dL) to assess locomotor activity before and after treatment. A group of 5 normal rats (NC) served as controls throughout the study. Rats were sacrificed, and the striatum was removed for biochemical and histological studies. In untreated diabetic rats, fructose/STZ administration resulted in hyperglycemia that altered locomotor function as characterized by increased freezing time, decreased mobility time, number of lines crossed, and total travel time compared to NC. MDA, TNF-*α*, INF-*γ*, and nitrite levels were elevated in the striatum of diabetic rats, while catalase activity and GSH levels were decreased, indicating oxidative stress and neuroinflammatory changes. In untreated diabetic rats, the microstructure of the HE-stained striatum revealed lipid vacuolation (hydropic degeneration) of the parenchyma, indicating a loss of neuronal integrity. The locomotor dysfunction was significantly improved by the aqueous extract of the SNP mixture, both biochemically and histologically. As a result, our findings support the mixture's ability to correct diabetes-related locomotion disorders as a glucose-lowering product and antioxidant, anti-inflammatory, and neuroprotective agent. These results justify the use of the aqueous extract of a combination of these three plants to manage diabetes and neuroinflammatory complications in Northern Cameroon.

## 1. Introduction

The prevalence of diabetes is increasing worldwide. It is one of the fastest-growing global health emergencies of the 21st century. The International Diabetes Federation (IDF) estimates that 536.6 million people live with diabetes (diagnosed or undiagnosed) in 2021, and this number is projected to increase by 46%, reaching 783.2 million by 2045 [1]. Diabetes mellitus is a heterogeneous metabolic disorder characterized by chronic hyperglycemia due to impaired insulin secretion, defective insulin action, or both [2]. Type 2 diabetes is the most common form of this disease, accounting for approximately 90% of diagnosed cases [2], and it has been consistently reported as one of the strongest correlates of mobility limitation, especially in older people, and a potential risk factor for future mobility disability and loss of independence [3]. Long-term type 2 diabetes results in many secondary complications that could negatively affect the structure and function of many organs including the eyes, kidneys, blood vessels, heart, and the central nervous system (CNS) [4]. Manifestations of diabetes-induced CNS alterations may include structural modifications leading to brain atrophy, as well as changes in electrophysiological properties that ultimately result in deficits in cognitive and locomotor performances [5] and a higher risk for dementia [6]. Striatopathy is a rare diabetic complication clinically manifested by hemichorea, an involuntary, continuous, non-patterned, irregular, and non-rhythmic movement involving one side of the body due to lesions found in the contralateral striatum [7, 8]. The pathophysiology of this condition is not fully understood. However, it is known that hyperviscosity due to hyperglycemia disrupts the blood-brain barrier which leads to vascular compromise of the striatal neurons [9], producing a synergistic effect of uncontrolled hyperglycemia and vascular damage causing dysfunction of the striatum leading to irregular movements [10]. Histopathology findings have shown gliosis and neuronal loss without evidence of infarction or hemorrhage within the striatum [10]. Studies have also reported an alteration of two inhibitory neurotransmitters: gamma aminobutyric acid (GABA) or dopamine [11].

Oxidative stress and inflammation have been involved in the past two decades as the main casual factors in the genesis of various chronic diseases and degenerative complications (diabetes, atherosclerosis, Parkinson's disease, and Alzheimer's disease). The brain is especially vulnerable to oxidative damage as a result of its high rate of oxygen consumption, abundant lipid content, and relative paucity of antioxidant enzymes compared to other tissues [12]. Therefore, neurons are particularly susceptible to oxidative damage, aging, and age-related diseases. These conditions disrupt the balance between reactive oxygen species (ROS) generation and antioxidant defense, which result in the degradation of biological macromolecules [13]. The resulting oxidative state causes neuroinflammation, damaging neuronal connections [14]. ROS also increases inflammation by activating certain stress-activated kinases and stimulates transcription factors such as nuclear factor-kappa B (NFκB) and activator protein-1 (AP-1), to trigger pro-inflammatory cytokines expression such as tumor necrosis factor (TNF-*α*), interleukin-1*β* (IL-1*β*), IL-6, and interferon gamma (INF-*γ*) [15].

As the first-line drug in the treatment of type 2 diabetes, biguanides, with metformin as the main representative, have been used to improve hyperglycemia in diabetic patients, thus avoiding diabetes-related CNS complications, especially cognitive decline [16, 17], but no clear evidence of their effectiveness in managing diabetes-associated locomotor disabilities has been reported. Furthermore, long-term use of this drug may result in a variety of serious adverse effects. Nowadays, strategies employing synergistic actions of medicinal plants may provide novel alternative therapies for diabetes-associated locomotor dysfunction.

*Sclerocarya birrea* and *Nauclea latifolia* stem barks and *Piper longum* fruits (SNP) are plants empirically used as macerated mixture by local populations in the Far North Region of Cameroon for the treatment of diabetes. Each of these plants has exhibited potent pharmacological activities. *Sclerocarya birrea* (Anacardiaceae), also called African yellow plum, is a medicinal plant used for the treatment of several diseases such as epilepsy, hypertension, inflammation, and memory disorders in Cameroonian folk medicine [18]. Studies conducted on the methanolic extract of *Sclerocarya birrea* stem bark have demonstrated its hypoglycemic properties [19]. Secondary metabolites contained in the stem bark were shown to be responsible for its antioxidant and antihyperglycemic properties [19, 20]. *Nauclea latifolia* (Rubiaceae) is used by traditional practitioners as a remedy for many diseases such as malaria, fever, diabetes, high blood pressure, and abdominal pain. The aqueous extract of *Nauclea latifolia* has been reported to have hypoglycemic, antihyperglycemic, anticonvulsive, and anxiolytic effects [21, 22]. *Piper longum* (Piperaceae) has been shown to have antihyperlipidemic, antidiabetic, antibacterial, antidepressant, hepatoprotective, anti-inflammatory, and cardioprotective properties [23, 24].

Although the mixture of these three macerated plants is empirically consumed by local populations, no scientific investigation has been done to validate its efficacy in resolving diabetes-related locomotor disabilities to the best of our knowledge. As a result, the purpose of this study is to investigate the efficacy of the SNP mixture on type 2 diabetes-induced locomotor abnormalities.

## 2. Materials and Methods

### 2.1. Plant Material Preparation

The stem barks of *Sclerocarya birrea* and *Nauclea latifolia* and fruits of *Piper longum* were harvested in Cameroon (Maga district, Far North Region, Cameroon, January 2019). Each plant was identified and authenticated by herbalist at the National Herbarium of Cameroon in comparison with different specimens deposited under the voucher numbers: No7770/SRF/CAM, No47652/HNC, and No34187/HNC, respectively, for *Sclerocarya birrea*, *Nauclea latifolia*, and *Piper longum*. The aqueous extract of the mixture of plant parts was prepared according to the traditional healer's recommendations. In brief, different parts of fresh plants were cleaned, cut into small pieces, dried in shade at room temperature, and then powdered to produce 200 g of *Sclerocarya birrea* powder, 200 g of *Nauclea latifolia* powder, and 90 g of *Piper longum* powder. The plant powders were mixed and macerated in 10 L of distilled water for 48 hours, filtered, and dried in a thermostat oven at 45°C. This allowed the acquisition of a raw dry extract (3.4 g), yielding 6.93% (w/w). The extract was then stored at room temperature in airtight boxes for further experiments.

### 2.2. Chemicals and Reagents

Fructose was purchased from Markal-ZA (France) and metformin tablets (Glucophage) from Merck S.L. Poligono (Barcelona, Spain). Streptozotocin (STZ) and analytical kits for TNF-*α* and INF-*γ* through rat enzyme-linked immunosorbent assay kit analysis were purchased from Sigma-Aldrich (St. Louis, MO, USA). Trichloroacetic acid, thiobarbituric acid, glacial acetic acid, ether, and formalin were also purchased from Sigma-Aldrich. Ellman and Griess reagents were obtained from Sigma-Aldrich and were freshly prepared before use. Drug solutions were prepared fresh and administered *per os* (po) in a volume of 10 mL/kg body weight (bw), except STZ which was administered intravenously (iv) at 1 mL/kg b.w.

### 2.3. Animals

Male Wistar rats aged 45 to 60 days (weighing 150–180 g) were raised in the animal facilities of the Laboratory of Animal Physiology (University of Yaoundé I, Cameroon) under standard light (12-hour day/night cycle), with access to the standard animal diet and tap water ad libitum during the trial. The animals were housed in groups of 5 rats per cage (40 cm × 40 cm). All experiments followed the principles of laboratory animal use and care of the “European Community Guidelines” (EEC Directive 2010/63/EEC) and were approved by the “Animal Ethical Committee” of the Faculty of Science, University of Yaoundé I.

### 2.4. Experimental Induction of Type 2 Diabetes Mellitus

Type 2 diabetes (T2D) induction was achieved using Sadeghi et al.'s slightly modified method [25]. In brief, animals received fructose (10%) orally in drinking water for 6 weeks and streptozotocin (35 mg/kg, *i.v.*). To prevent death from STZ-induced hypoglycemic shock, rats were given 10% glucose solution for 24 hours after STZ injection. A group of normal rats receiving NaCl (0.9%, *i.v.*) and tap water orally was added during the trial. Two weeks after glycemia stability, the fasting glucose level (FGL) was measured in blood collected from the tail of STZ-treated rats, and animals showing a FGL between 126 and 350 mg/dL were considered diabetic. The FGL was determined using a portable glucometer (Accu-Check Active, Roche Diagnostic Corporation, Germany). After that, diabetic rats were maintained for four additional weeks to allow the development of diabetes complications. At the end of this period, all rats were subjected to the open field test for 10 min to assess locomotor activity.

### 2.5. Evaluation of the Activity of SNP Mixture against T2D-Induced Locomotion Disorders in Diabetic Rats

**Figure 1:**
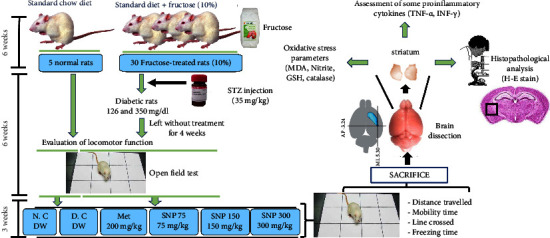
Diagram of the experimental protocol. N.C = normal control; D.C = diabetic control; Met = metformin; SNP 75, SNP 150, and SNP 300 = *Sclerocarya birrea*, *Nauclea latifolia*, and *Piper longum* mixture aqueous extract at doses of 75, 150, and 300 mg/kg; STZ = streptozotocin; DW = distilled water (10 mL/kg); MDA = malondialdehyde; GSH = reduced glutathione; TNF-*α* = tumor necrosis factor alpha; INF-*γ* = interferon gamma; H-E = hematoxylin-eosin.

A total of 30 rats (5 non-diabetics and 25 diabetics) were grouped as follows: a normal control group (non-diabetic rats), treated orally with distilled water (DW), a diabetic control group treated with DW, a positive control group (Met 200) receiving metformin (200 mg/kg, *p.o.*), and three groups treated orally with SNP mixture at doses of 75 mg/kg (SNP 75), 150 mg/kg (SNP 150), and 300 mg/kg (SNP 300). Treatment was administered once a day to the animals for 21 consecutive days, and FGL was monitored every week. Every three days, animals' body weight was measured. At the end of the experimental period, animals' locomotor activity using open field test was assessed. The animals were then sacrificed under anesthesia (ether), and the striatum was extracted from the brains for biochemical and histological analysis. The experimental protocol is shown in Figure 1.

### 2.6. Behavior Test (Open Field Test)

Open field testing (OFT) was conducted before and after the treatment period (between 20:00 and 23:00) in a quiet environment to observe animal behavior and locomotion. This test was carried out at night due to intense animal activity [26]. The OFT was conducted as described in [27]. The test environment was an open field constructed of wood material (80 cm length × 80 cm width × 60 cm height) with a white floor divided into squares of 10 cm length. In the test, rats were introduced to the apparatus (an unfamiliar arena) and given 10 minutes to explore it. Locomotor activity was determined by measuring the distance traveled (m), mobility time (s), number of lines crossed, and freezing time (s). All rat movements were recorded and automatically analyzed by ANY-maze 7.15 video tracking system (Stoelting, Wood Dale, IL, USA) with a Logitech high-resolution camera [28].

### 2.7. Biochemical Analysis

#### 2.7.1. Brain Dissection and Homogenization

Following the behavioral assessment, animals were immediately sacrificed by decapitation under ether anesthesia. The brain of each rat was rapidly removed and divided into two hemispheres. The striatum was isolated from one hemisphere, washed in ice-cold saline, and blotted to dryness. Then, they were homogenized in cold Tris-HCl buffer (pH = 7.4) in a ratio of 20% (w/v). After centrifugation of each homogenate at 3000 rpm and 4°C for 25 min, the supernatant was collected into tubes and stored at −20°C for further biochemical evaluation. The other hemisphere of the brain was fixed in neutral buffered formalin (4%) for later histological analysis. As a result of the high blood sugar levels achieved in animals treated with the extract at 75 mg/kg, their reduced body weight at the end of the treatment period, and the locomotor activity, the group treated with the extract (75 mg/kg) was removed from biochemical and histological studies.

#### 2.7.2. Levels of Pro-Inflammatory Marker

TNF-*α* and INF-*γ* levels were determined using rat enzyme-linked immunosorbent assay (ELISA) kits as directed by the manufacturer. In brief, fifty microliters of the standard or sample were added to a corresponding well precoated with TNF-*α* or INF-*γ* specific antibodies for rats. The mixture was gently mixed for 10 seconds and incubated for 30 minutes at room temperature. Wells were washed three times (with a washer buffer provided by the manufacturer). In each well, 100 *μ*L of horseradish peroxidase-conjugated horseradish specific antibodies (anti-TNF-*α* or anti-INF-*γ*) conjugated to horseradish peroxidase was added and the mixture was incubated for two hours. The preparation was then washed three times and incubated with streptavidin for 30 minutes. The enzyme reaction was stopped using a sulfuric acid solution. The optical density of each well was measured using a microplate reader. The concentration of each cytokine was estimated from the corresponding calibration curve.

#### 2.7.3. Oxidative and Nitrative Stress Markers

The Wilbur method was used to calculate malondialdehyde (MDA) in striatum homogenate. The MDA content was measured at 530 nm against the blank after its reaction with trichloroacetic acid and thiobarbituric acid. MDA concentration was measured in *μ*mol per g of organ [29]. Nitric levels were estimated by measuring the amount of nitrite using the Griess reagent. 50 *μ*L of homogenate and 200 *μ*L of distilled water were mixed in tubes, and 250 *μ*L of Griess reagent (0.1% naphthyl ethylenediamine, 1% sulfanilamide, and 5% phosphoric acid) was added, and the absorbance was measured at 570 nm against the blank [30]. The amount of nitrite was estimated in *μ*mol per g of organ.

#### 2.7.4. Endogenous Antioxidants

The concentration of reduced glutathione (GSH) was determined using the Ellman method [31]. The GSH content was measured at 412 nm against the blank after its reaction with Ellman reagent (dinitro-2,2′-dithio-5,5′-dinitrobenzoic acid). The amount of GSH was expressed in *μ*mol per g of organ. Catalase activity was measured using the Sinha technique [32], based on the principle of hydrogen peroxide (H_2_O_2_) degradation in the presence of the catalase enzyme. The concentration of undecomposed H_2_O_2_ was evaluated using a calibration curve established from a standard solution (50 mM H_2_O_2_). Tissue catalase activity was represented as mM H_2_O_2_ per g of organ.

### 2.8. Histological Analysis

The half brains were used for routine staining (HE stain). Each brain sections were dehydrated in alcohol and embedded in paraffin blocks, and tissue sections with 5 *μ*m thickness were stained with hematoxylin and eosin (HE) [33]. The stained and mounted slides were observed at different magnifications (×40 and ×250) using a Scientico STM-50 optical microscope (HSIDC Industrial Estate, Haryana, India) equipped with a Celestron 44421 digital camera connected to a computer (HP Pavilion, g series).

### 2.9. Statistical Analysis

The results are presented as mean ± standard error of the mean (SEM). Statistical analysis and presentation of data were performed using GraphPad Prism (version 8.00; GraphPad Software, Inc., San Diego, CA, USA). Data were analyzed using one-way analysis of variance (ANOVA) followed by the Tukey–Kramer multiple comparison test. Student's *t*-test was applied to analyze behavioral data between normal and diabetic control before treatment. *P* values less than 0.05 implied statistical difference.

## 3. Results

### 3.1. Effects of Fructose/STZ-Induced Type 2 Diabetes on Blood Glucose Level and Glucose-Lowering Properties of *Sclerocarya birrea*, *Nauclea latifolia*, and *Piper longum* Mixture Aqueous Extract

**Figure 2:**
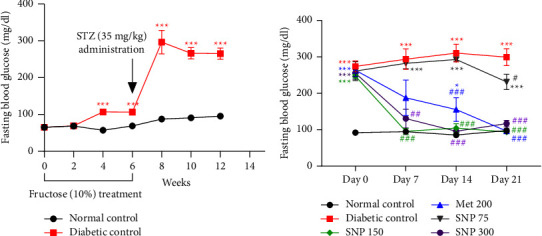
Effects of fructose/STZ-induced type 2 diabetes on blood glucose level (a) and glucose-lowering properties of *Sclerocarya birrea*, *Nauclea latifolia,* and *Piper longum* (SNP) mixture aqueous extract (b). Data are shown as mean ± SEM, 20 ≤ *n* ≥ 5 in (a) and *n* = 5 in (b); ^*∗*^*P* < 0.05 and ^*∗∗∗*^*P* < 0.001: significant as compared to normal control; ^##^*P* < 0.01 and ^###^*P* < 0.001: significant as compared to diabetic control; Met 200: diabetic animals receiving metformin at 200 mg/kg b.w.; SNP 150, 300: diabetic animals receiving the aqueous extract of the mixture at 150 and 300 mg/kg b.w.

Figure 2(a) shows the change in blood glucose levels during the induction phase. Following fructose ingestion and STZ injection, blood glucose levels increased dramatically and remained elevated for six weeks (FBG between 265 and 296 mg/dL from week eight to week twelve). During this period, diabetic rats had considerably increased FBG (*P* < 0.001) than normal controls. Likewise, FBG from diabetic animals remained considerably higher (*P* < 0.001) between 273 and 311 mg/dL during the treatment period, as illustrated in Figure 2(b). Treatment with the extract at doses of 150 and 300 mg/kg caused a progressive drop in blood sugar levels. Compared to the diabetic control, the extract at the dose level elicited a substantial decrease (*P* < 0.001) in blood glucose levels of 67.7%, 66.2%, and 68.6%, respectively, on days 7, 14, and 21. Similarly, the extract at 300 mg/kg dosing level reduced blood glucose levels by 55.4% (*P* < 0.01), 66.2%, and 68.6% (*P* < 0.001) on days 7, 14, and 21, respectively, as compared to the diabetic control. Likewise, metformin treatment resulted in a significant drop (*P* < 0.001) in blood glucose levels on days 14 and 21, respectively. In contrast, the plant mixture at the dose of 75 mg/kg did not reduce blood glucose levels during the treatment period, except on day 21, when a modest decrease was observed (*P* < 0.05) when compared to the diabetic control.

### 3.2. Effects of the Aqueous Extract of *Sclerocarya birrea*, *Nauclea latifolia*, and *Piper longum* Mixture on Body Weight Changes in Diabetic Rats

**Figure 3:**
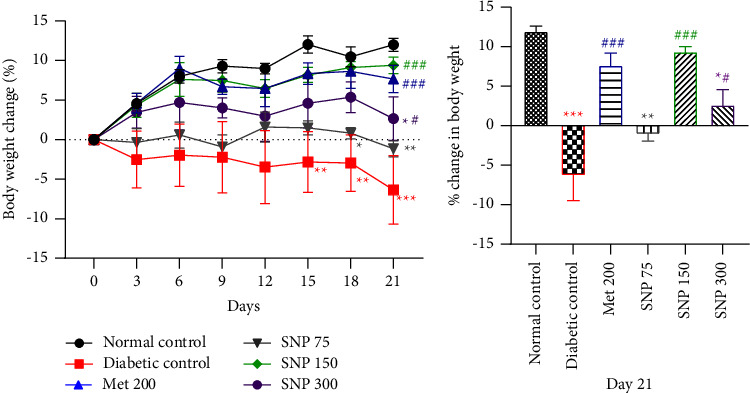
Evolution in body weight throughout the experimental period (a) and percentage change in body weight in diabetic rats (b). Data are shown as mean ± SEM, *n* = 5; ^*∗*^*P* < 0.05, ^*∗∗*^*P* < 0.01, and ^*∗∗∗*^*P* < 0.001: significant as compared to normal control; ^#^*P* < 0.05, ^##^*P* < 0.01, and ^###^*P* < 0.001: significant as compared to diabetic control; Met 200: diabetic animals receiving metformin at 200 mg/kg b.w.; SNP 150, 300: diabetic animals receiving the aqueous extract of the mixture at 150 and 300 mg/kg b.w.

Fructose ingestion followed by STZ administration led to a gradual loss of body weight in diabetic control throughout the treatment period, as shown in Figure 2(a). There was a significant decrease in body weight on days 15, 18 (*P* < 0.01), and 21 (*P* < 0.001) compared to the normal control. As shown in Figures 3(a) and 3(b), the plant mixture administered for 21 days significantly increased body weight on day 21 at doses of 150 mg/kg (*P* < 0.001) and 300 mg/kg (*P* < 0.05) when compared to diabetic rats. Similarly, metformin-treated rats showed a significantly increased body weight (*P* < 0.001). On the other hand, the plant mixture at the dose of 75 mg/kg did not improve body weight loss throughout the experimental period (Figure 3(b)). Moreover, extract-treated animals at 75 mg/kg failed to improve this parmeter when compared to untreated diabetic rats.

### 3.3. Effects of *Sclerocarya birrea*, *Nauclea latifolia*, and *Piper longum* Mixture Aqueous Extract on Spontaneous Locomotion in Diabetic Rats

**Figure 4:**
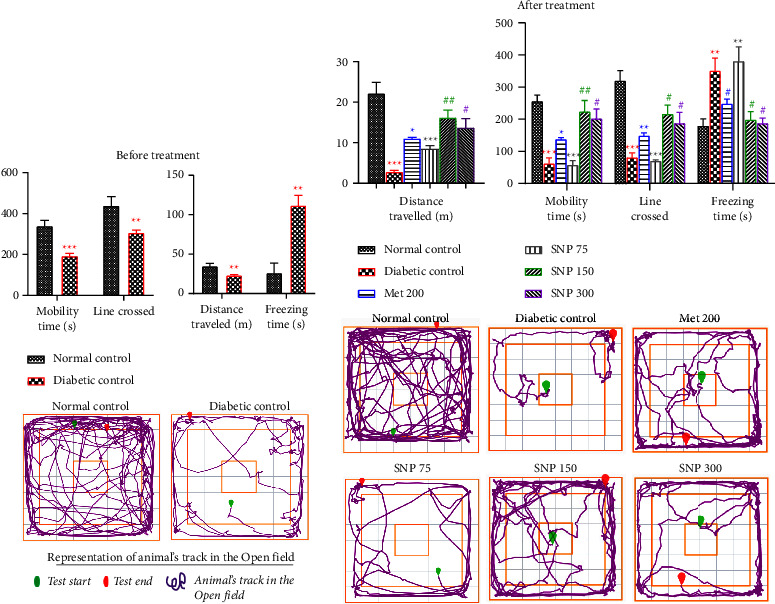
Effects of fructose/STZ-induced type 2 diabetes on spontaneous locomotion (a) and effects of the aqueous extract of the SNP mixture on spontaneous locomotion in T2D rats (b). Data are shown as mean ± SEM, 20 ≤ *n* ≥ 5 in (a) and *n* = 5 in (b); ^*∗∗*^*P* < 0.01 and ^*∗∗∗*^*P* < 0.001: significant as compared to normal control; ^#^*P* < 0.05 and ^##^*P* < 0.01: significant as compared to diabetic control; Met 200: diabetic animals receiving metformin at 200 mg/kg b.w.; SNP 150, 300: diabetic animals receiving the aqueous extract of the mixture at 150 and 300 mg/kg b.w.

Diabetic animals in the open field test exhibited a significant decrease in total mobility time (*P* < 0.001), total traveled distance (*P* < 0.01), and number of lines crossed (*P* < 0.01), while freezing time significantly (*P* < 0.01) rose six weeks post-STZ as compared to the normal control group (Figure 4(a)). These results were observable in the diagram representing the animal's track during the test where a very low activity of diabetic animals was noted when compared to the activity of normal animals. In Figure 4(b), it is discernible that untreated diabetic rats showed a marked decrease in total mobility time (*P* < 0.01), total travel distance (*P* < 0.001), and number of crossed lines crossed (*P* < 0.001) and a significant increase in freezing time (*P* < 0.01) as compared to the normal control. The administration of the extract for three weeks at a dose of 150 mg/kg significantly increased the total mobility time (*P* < 0.01), total distance traveled (*P* < 0.01), number of lines crossed (*P* < 0.05) and the decrease in freezing time (*P* < 0.05) compared to the diabetic control. A similar result was observed in animals treated with the extract at 300 mg/kg dose level. However, when compared to the normal control, animals treated with the extract at the dose of 75 mg/kg showed a significant increase (*P* < 0.01) in freezing time and a significant decrease (*P* < 0.00*1*) in total mobility time, total distance traveled, and the number of lines crossed, similar to the diabetic control group results.

### 3.4. Effects of the Aqueous Extract of *Sclerocarya birrea*, *Nauclea latifolia*, and *Piper longum* Mixture on TNF-*α* and INF-*γ* Levels in the Striatum of Diabetic Rats

**Figure 5:**
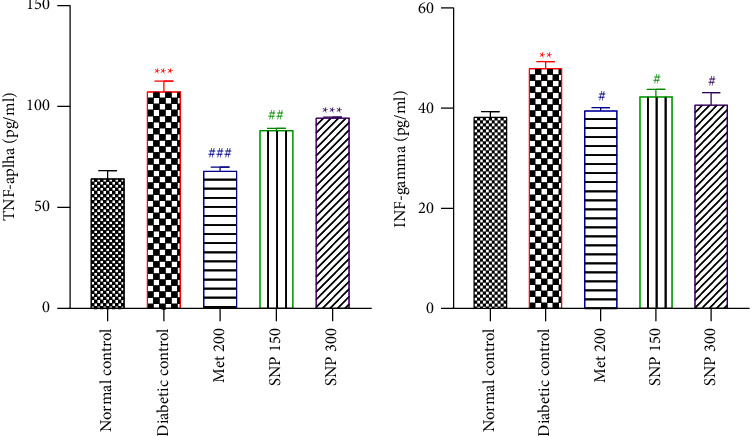
Effects of the aqueous extract of the SNP mixture on TNF-*α* (a) and INF-*γ* (b) levels in the striatum of T2D rats. Data are shown as mean ± SEM, *n* = 5; ^*∗∗*^*P* < 0.01 and ^*∗∗∗*^*P* < 0.001: significant as compared to normal control; ^#^*P* < 0.05, ^##^*P* < 0.01, and ^###^*P* < 0.001: significant as compared to diabetic control. Met 200: diabetic animals receiving metformin at 200 mg/kg b.w.; SNP 150, 300: diabetic animals receiving the aqueous extract of the mixture at 150 and 300 mg/kg b.w.

Figure 5 represents the effects of the aqueous extract of the SNP mixture on striatum levels of TNF-*α* (Figure 5(a)) and INF-*γ* (Figure 5(b)). Fructose/STZ-induced diabetes resulted in a significant increase in TNF-*α* (*P* < 0.001) and INF-*γ* (*P* < 0.01) levels in diabetic control compared to normal control. Compared to the diabetic control, administration of the SNP combination at a dose of 150 mg/kg significantly lowered TNF-*α* (*P* < *0.01*) and INF-*γ* (*P* < 0.05) levels. However, at a dose of 300 mg/kg, the extract did not diminish TNF-*α* levels while dramatically lowering INF-*γ* levels (*P* < 0.05). The metformin-treated group presented a marked decrease in TNF-*α* (*P* < 0.001) and INF-*γ* (*P* < 0.05) levels.

### 3.5. Effects of the Aqueous Extract of Sclerocarya birrea, Nauclea latifolia, and Piper longum Mixture on MDA and Nitrites Levels in the Striatum of Diabetic Rats

**Figure 6:**
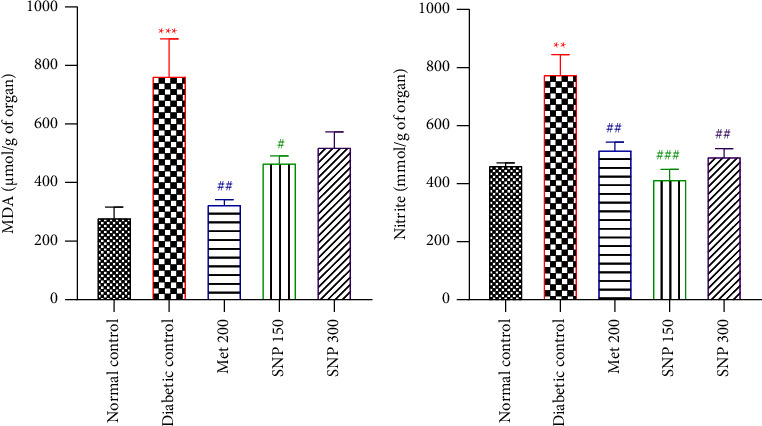
Effects of SNP mixture aqueous extract on MDA (a) and nitrite (b) levels in the striatum of T2D rats. Data are shown as mean ± SEM, *n* = 5; ^*∗∗*^*P* < 0.01 and ^*∗∗∗*^*P* < 0.001: significant as compared to normal control; ^#^*P* < 0.05, ^##^*P* < 0.01, and ^###^*P* < 0.001: significant as compared to diabetic control. Met 200: diabetic animals receiving metformin at 200 mg/kg b.w.; SNP 150, 300: diabetic animals receiving the aqueous extract of the mixture at 150 and 300 mg/kg b.w.

Fructose/STZ-induced diabetes resulted in significantly increased levels of MDA (*P* < 0.001) and nitrite (*P* < 0.01) levels in the striatum of untreated diabetic rats compared to normal rats (Figures 6(a) and 6(b)). The extract significantly reduced striatal MDA levels at the doses of 150 mg/kg (*P* < 0.001) and 300 mg/kg (*P* < 0.01) levels at the end of the treatment period. Extract-treated and metformin groups presented a significant (*P* < 0.01 − 0.001) drop in striatal nitrite levels.

### 3.6. Effects of the Aqueous Extract of Sclerocarya birrea, Nauclea latifolia, and Piper longum Mixture on Glutathione Levels and Catalase Activity in the Striatum of Diabetic Rats

**Figure 7:**
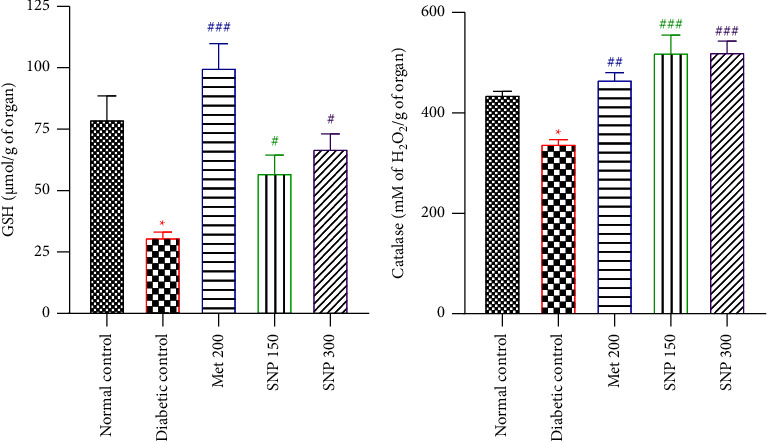
Effects of the aqueous extract of the SNP mixture on GSH level (a) and catalase activity (b) in the striatum of T2D rats. Data are shown as mean ± SEM, *n* = 5; ^*∗*^*P* < 0.05 and ^*∗∗∗*^*P* < 0.001: significant as compared to normal control; ^#^*P* < 0.05, ^##^*P* < 0.01, and ^###^*P* < 0.001: significant as compared to diabetic control. Met 200: diabetic animals receiving metformin at 200 mg/kg b.w.; SNP 150, 300: diabetic animals receiving the aqueous extract of the mixture at 150 and 300 mg/kg b.w.

Fructose/STZ-induced diabetes resulted in significantly reduced (*P* < 0.05) glutathione levels and catalase activity in the striatum of untreated diabetic rats as compared to normal rats (Figures 7(a) and 7(b)). Following the 21-day treatment period with the aqueous extract at both doses, striatum GSH levels increased significantly (*P* < 0.05) as well as catalase activity (*P* < 0.001) when compared to the diabetic control. Similarly, a marked increase in GSH levels (*P* < 0.001) and catalase activity (*P* < 0.01) was observed in the metformin-treated group.

### 3.7. Effects of the Aqueous Extract of *Sclerocarya birrea*, *Nauclea latifolia*, and *Piper longum* Mixture on Striatum's Microarchitecture in Diabetic Rats

**Figure 8:**
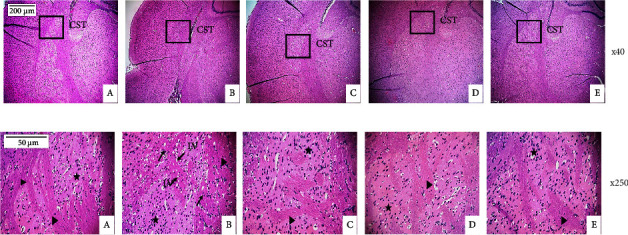
Microphotographs of the H&E-stained striatum on the caudal axis in diabetic rats ((a) ×40 and (b) ×250). Tail striatum outlines are indicated by black squares (×40). CST: caudal striatum; LV: lipid vacuolation (arrows); bundles of nigrostriatal tract (arrowhead); small neurons (stars); (A) normal control; (B) diabetic control; (C) Met 200; (D) SNP 150; (E) SNP 300. Met 200: diabetic animals receiving metformin at 200 mg/kg b.w.; SNP 150, 300: diabetic animals receiving the aqueous extract of the mixture at 150 and 300 mg/kg b.w.

Figure 8 shows the effect of the aqueous extract of *Sclerocarya birrea*, *Nauclea latifolia*, and *Piper longum* mixture on the microstructure of the striatum. Diabetes provoked the development of lipid vacuolations (hydropic degeneration) in the parenchyma of untreated diabetic rats, demonstrating a loss of the integrity of the neuronal tissue (Figure 8(b)). In contrast to diabetic rats that did not receive treatment, these changes were reversed by the extract at doses of 150 and 300 mg/kg as well as metformin-treated group.

## 4. Discussion

In the present study, the effect of the aqueous extract of the mixture of *Sclerocarya birrea* and *Nauclea latifolia* stem barks and *Piper longum* fruits on type 2 diabetes-induced locomotor dysfunction was investigated. Fructose (10%) ingestion for 6 weeks followed by streptozotocin administration (35 mg/kg) resulted in high blood glucose levels which altered locomotor function and increased oxidative and nitrative stress and inflammation in the striatum. A combination of fructose (10%) and low-dose STZ (35 mg/kg) is a well-known alternative model to induce major pathogeneses of T2D, insulin resistance, and partial pancreatic *β*-cell dysfunction in rats [34, 35]. This model more closely resembles the later stages of T2D and has demonstrated a stable diabetic condition over an 11-week experimental period useful for chronic research studies as well as for routine pharmacological screening [35, 36]. In the current study, diabetes was confirmed by the recorded increase in blood glucose throughout the experiment. This result could be explained by the fructose pretreatment coupled with injected streptozotocin which has destructed pancreatic *β* cells and led to impaired glucose-stimulated insulin release and insulin resistance. Thus, the subsequent elevation of blood glucose may be attributed to the reduced entry of glucose into peripheral tissues, muscle, and adipose tissue, increasing glycogen breakdown, gluconeogenesis, and hepatic glucose production [36, 37]. Over time, this condition significantly increased blood glucose level, and the resulting hyperglycemia leads to hyperviscosity of the blood disrupting the blood-brain barrier, leading to vascular compromise of the striatal neurons [9]. The synergistic effect of uncontrolled hyperglycemia and vascular destitution caused a dysfunction of the striatum leading to irregular movements [10]. This phenomenon is accompanied by the release of reactive oxygen species (ROS), nitric oxide, and pro-inflammatory cytokines such as interleukin IL-1*β*, IL-6, INF-*γ*, and TNF-*α* in the brain [13, 14], and the striatum is not spared. In our study, untreated diabetic rats presented a significantly elevated blood glucose level and marked increase in MDA, NO, TNF-*α*, and INF-*γ* levels in the striatum. The striatum damages attributed to chronic hyperglycemia were histologically displayed in the diabetic control by the presence of lipid vacuolation (hydropic degeneration) showing a loss of integrity of neuronal tissue (Figure 8). The increased production of reactive oxygen and nitrogen species caused by prolonged hyperglycemic conditions may disrupt the blood-brain barrier, leading to vascular compromise of the functioning of striatal neurons and their death [38–40]. Treatment with the combination of plants, especially at the dose of 150 mg/kg, has significantly reduced blood glucose levels (*P* < 0.001) and decreased oxidative (MDA) and nitrative (NO) stress markers levels and pro-inflammatory cytokines levels (INF-*γ* and TNF-*α*) in the striatum. These results were confirmed histologically in the striatum of extract-treated groups, where normal parenchyma was noted, thus suggesting that the aqueous extract of the SNP mixture could restore striatum damage attributed to diabetes. This result could be explained by the synergistic effect of secondary metabolites (mainly flavonoids, polyphenols, alkaloids, and several other compounds (JPDT and FNT, University of Yaoundé 1, Cameroon, Unpublished results)) that are abundant in the plant mixture that inhibits or eliminates ROS, NO, and pro-inflammatory cytokine release, thus preventing the hyperglycemia-induced neuronal damage and protecting striatum neurons from the morbid effect. In fact, these plant-based compounds have been proven to possess neuroprotective, antioxidant, anti-inflammatory, and antidiabetic properties [19, 20, 22, 41].

Chronic long-term diabetes complications have been associated with the pathogenesis of muscle impairment in type 2 diabetic patients [42]. Diabetes-related muscle weakness is closely related to reduced body weight and may result in impaired mobility. We found a significant drop in body weight in untreated diabetic rats. Long-term hyperglycemia, oxidative stress, and chronic inflammation are all important factors in the development of diabetes complications [42]. These factors can disrupt the balance of protein synthesis and breakdown, cause mitochondrial dysfunction, and induce apoptosis, resulting in fiber atrophy and loss, decreased body weight, and eventually sarcopenia. The combined effects of this phenomenon and striatum damage severely limit the physical activities of diabetic rats, leading to locomotor disorders. Both doses of SNP mixture improved body weight changes in diabetic rats, with a significant increase at 150 mg/kg on day 21 (*P* < 0.001). Rats treated with metformin had similar outcomes. These findings show that in addition to protecting striatal neurons, the SNP mixture improves body weight changes and muscle strength by lowering hyperglycemia, oxidative stress, and inflammation. The improvement in locomotor activity was justified in the OFT. In fact, these results highlight a significant decrease in total mobility time (*P* < 0.001), total traveled distance (*P* < 0.01), and number of lines crossed (*P* < 0.01) and a significant increase in freezing time (*P* < 0.01) in untreated diabetic rats when compared to normal rats. These results agree with other studies that showed impaired locomotor activity in diabetic rats [27, 43]. Rats treated with the SNP mixture at both doses traveled longer distances and were mobile and active compared to untreated diabetic rats. These results indicate that the aqueous extract of the SNP mixture could improve locomotor function under diabetic conditions, as it contains a number of synergistic active metabolites. Studies have shown that the striatum is involved in both movement generation and inhibition [44]. Two distinct classes of pathway projection neurons responsible for the control of locomotion have been highlighted in the striatum: direct pathway striatal neurons that facilitate motor output (movement) and indirect pathway neurons that inhibit motor output [44]. The expression of each pathway is modulated by dopamine through action on its receptors. Dopamine Drd1 receptors are selectively expressed by direct pathway neurons, whereas dopamine Drd2 receptors are selectively expressed by indirect pathway neurons [44]. In the initiation of movement, direct pathway medium spiny neurons project to the internal globus pallidus and substantia nigra pars reticulata, whereas in the inhibition of movement, indirect pathway medium spiny neurons express project indirectly to the substantia nigra pars reticulata by way of the external globus pallidus and subthalamic nucleus [44, 45]. In the current study, it is believed that diabetes, through increased production of ROS, nitric oxide, and pro-inflammatory cytokines, can promote the indirect pathway through activation of the dopamine Drd2 receptor to inhibit motor output, and that the SNP mixture may lessen this effect while stimulating direct pathway striatal neurons through activation of the dopamine Drd1 receptor, further improving locomotion in diabetic rats. To confirm this hypothesis, further experiments are needed. However, this study confirms the capacity of the SNP mixture as an antidiabetic, antioxidant, anti-inflammatory, and neuroprotective agent to correct locomotor disorders and protect striatal neurons in diabetic rats.

## 5. Conclusion

In the study carried out, the efficacy of the aqueous extract of the SNP mixture on type 2 diabetes-induced locomotor abnormalities was examined. Our findings imply that the aqueous extract of the SNP mixture may protect striatal neurons from chronic hyperglycemia-induced neuronal damage and improve locomotion in diabetic rats. The ability of the plant mixture to reduce blood sugar and have antioxidant and anti-inflammatory activities may explain its neuroprotective efficacy. Additionally, our findings may provide new avenues for investigating the plant mixture's underlying mechanisms for the therapy of movement disorders associated with diabetes. Therefore, more research is required to shed light on the potential processes through which SNP mixture improves locomotor function in type 2 diabetic rats.

## Data Availability

The data used to support the findings of this study are available from the corresponding author upon reasonable request.

## References

[1] Sun H., Saeedi P., Karuranga S. (2022). IDF Diabetes Atlas: global, regional and country-level diabetes prevalence estimates for 2021 and projections for 2045. *Diabetes Research and Clinical Practice*.

[2] Punthakee Z., Goldenberg R., Katz P. (2018). Definition, classification and diagnosis of diabetes, prediabetes and metabolic syndrome. *Canadian Journal of Diabetes*.

[3] Bianchi L., Zuliani G., Volpato S. (2013). Physical disability in the elderly with diabetes: epidemiology and mechanisms. *Current Diabetes Reports*.

[4] Lotfy M., Adeghate J., Kalasz H., Singh J., Adeghate E. (2016). Chronic complications of diabetes mellitus: a mini review. *Current Diabetes Reviews*.

[5] Gispen W. H., Biessels G. J. (2000). Cognition and synaptic plasticity in diabetes mellitus. *Trends in Neurosciences*.

[6] Degen C., Toro P., Schönknecht P., Sattler C., Schröder J. (2016). Diabetes mellitus Type II and cognitive capacity in healthy aging, mild cognitive impairment and Alzheimer’s disease. *Psychiatry Research*.

[7] Qi X., Yan Y. Y., Gao Y., Zheng Z. S., Chang Y. (2012). Hemichorea associated with non-ketotic hyperglycaemia: a case report. *Diabetes Research and Clinical Practice*.

[8] Shahzad W., Inayat T., Syed F. (2021). Diabetic striatopathy associated with type 2 diabetes: a rare complication. *Journal of Clinical and Translational Endocrinology: Case Reports*.

[9] Slabu H., Savedia-Cayabyab S., Senior P., Arnason T. (2011). Permanent haemichorea associated with transient hyperglycemia. *Case Reports*.

[10] Lin J. J. (2001). Ipsilateral putamen hyperintensity on T1-weighted MRI in non-ketotic hyperglycemia with hemiballism-hemichorea: a case report. *Parkinsonism & Related Disorders*.

[11] Battisti C., Forte F., Rubenni E. (2009). Two cases of hemichorea-hemiballism with nonketotic hyperglycemia: a new point of view. *Neurological Sciences*.

[12] Muriach M., Flores-Bellver M., Romero F. J., Barcia J. M. (2014). Diabetes and the brain: oxidative stress, inflammation, and autophagy. *Oxidative Medicine Cellular Longevity*.

[13] Butterfield D. A., Sultana R. (2008). Redox proteomics: understanding oxidative stress in the progression of age-related neurodegenerative disorders. *Expert Review of Proteomics*.

[14] Bhutada P., Mundhada Y., Bansod K. (2010). Ameliorative effect of quercetin on memory dysfunction in streptozotocin-induced diabetic rats. *Neurobiology of Learning and Memory*.

[15] Liu T., Zhang L., Joo D., Sun S. C. (2017). NF-*κ*B signaling in inflammation. *Signal Transduction and Targeted Therapy*.

[16] Areosa Sastre A., Vernooij R. W., González-Colaço Harmand M., Martínez G. (2017). Effect of the treatment of Type 2 diabetes mellitus on the development of cognitive impairment and dementia. *Cochrane Database of Systematic Reviews*.

[17] Samaras K., Makkar S., Crawford J. D. (2020). Metformin use is associated with slowed cognitive decline and reduced incident dementia in older adults with type 2 diabetes: the sydney memory and ageing study. *Diabetes Care*.

[18] Nonmarmbaye R., Kouemou N., Neteydji S., Njapdounke J. S. K., Bum E. N., Elisabeth N. B. (2021). Mnemonic and neuroprotective properties of Sclerocarya birrea root bark decoction on mouse model of monosodium glutamate-induced neurotoxicity involve by its antioxidant activities. *GSC Biological and Pharmaceutical Sciences*.

[19] Dimo T., Rakotonirina S. V., Tan P. V. (2007). Effect of Sclerocarya birrea (Anacardiaceae) stem bark methylene chloride/methanol extract on streptozotocin-diabetic rats. *Journal of Ethnopharmacology*.

[20] Chukwuma C. I., Matsabisa M. G., Ibrahim M. A., Erukainure O. L., Chabalala M. H., Islam M. S. (2019). Medicinal plants with concomitant anti-diabetic and anti-hypertensive effects as potential sources of dual acting therapies against diabetes and hypertension: a review. *Journal of Ethnopharmacology*.

[21] Gidado A., Ameh D. A., Atawodi S. E., Ibrahim S. (2008). Hypoglycaemic activity of Nauclea latifolia Sm. (Rubiaceae) in experimental animals. *African Journal of Traditional, Complementary and Alternative Medicines*.

[22] Ngo Bum E., Taiwe G. S., Moto F. C. (2009). Anticonvulsant, anxiolytic, and sedative properties of the roots of Nauclea latifolia Smith in mice. *Epilepsy and Behavior*.

[23] Kilari E. K., Rao L. S. N., Sreemanthula S., Kola P. K. (2015). Anti-stress and nootropic activity of aqueous extract of Piper longum fruit, estimated by noninvasive biomarkers and Y-maze test in rodents. *Environ Exp Biol*.

[24] Nabi S. A., Kasetti R. B., Sirasanagandla S., Tilak T. K., Kumar M. V. J., Rao C. A. (2013). Antidiabetic and antihyperlipidemic activity of Piper longum root aqueous extract in STZ induced diabetic rats. *BMC Complementary and Alternative Medicine*.

[25] Sadeghi A., Beigy M., Alizadeh S. (2017). Synergistic effects of ad-libitum low-dose fructose drinking and low-dose streptozotocin treatment in wistar rats: a mild model of type 2 diabetes. *Acta Medica Iranica*.

[26] Norton S., Culver B., Mullenix P. (1975). Development of nocturnal behavior in albino rats. *Behavioral Biology*.

[27] Bădescu S. V., Tătaru C. P., Kobylinska L. (2016). Effects of caffeine on locomotor activity in streptozotocin-induced diabetic rats. *Journal of medicine and life*.

[28] Zhou X., Wang S., Ding X. (2017). Zeaxanthin improves diabetes-induced cognitive deficit in rats through activiting PI3K/AKT signaling pathway. *Brain Research Bulletin*.

[29] Wilbur K. M., Bernheim F., Shapiro O. W. (1949). The thiobarbituric acid reagent as a test for the oxidation of unsaturated fatty acids by various agents. *Archives of Biochemistry*.

[30] Tsikas D. (2007). Analysis of nitrite and nitrate in biological fluids by assays based on the Griess reaction: appraisal of the Griess reaction in the L-arginine/nitric oxide area of research. *Journal of Chromatography B*.

[31] Ellman G. L. (1959). Tissue sulfhydryl groups. *Archives of Biochemistry and Biophysics*.

[32] Sinha A. K. (1972). Colorimetric assay of catalase. *Analytical Biochemistry*.

[33] Galba Jean B., Alice Irène F., Albert Donatien A. (2022). Neuroprotective effects of the ethanolic leaf extract of crassocephalum crepidioides (asteracaeae) on diazepam-induced amnesia in mice. *Advances in Pharmacological and Pharmaceutical Sciences*.

[34] Udumula M. P., Mangali S., Kalra J. (2021). High fructose and streptozotocin induced diabetic impairments are mitigated by Indirubin-3-hydrazone via downregulation of PKR pathway in Wistar rats. *Scientific Reports*.

[35] Wilson R. D., Islam M. S. J. P. R. (2012). Fructose-fed streptozotocin-injected rat: an alternative model for type 2 diabetes. *Pharmacological Reports*.

[36] Gheibi S., Kashfi K., Ghasemi A. (2017). A practical guide for induction of type-2 diabetes in rat: incorporating a high-fat diet and streptozotocin. *Biomedicine & Pharmacotherapy*.

[37] Bouhali I., Tayaa H., Tahraoui A. (2015). Quercetin, a natural flavonoid, mitigates fenthion induced locomotor impairments and brain acetylcholinesterase inhibition in male wistar rat. *Middle-East Journal of Scientific Research*.

[38] Higa M., Kaneko Y., Inokuchi T. (2004). Two cases of hyperglycemic chorea in diabetic patients. *Diabetic Medicine*.

[39] Lehner C., Gehwolf R., Tempfer H. (2011). Oxidative stress and blood-brain barrier dysfunction under particular consideration of matrix metalloproteinases. *Antioxidants and Redox Signaling*.

[40] Prasad S., Sajja R. K., Naik P., Cucullo L. (2014). Diabetes mellitus and blood-brain barrier dysfunction: an overview. *Journal of Pharmacovigilance and Drug Research*.

[41] Kumar S., Sharma S., Suman J. (2011). Vivo anti-hyperglycemic and antioxidant potential of Piper longum fruit. *Journal of Pharmaceutical Research*.

[42] Bianchi L., Volpato S. (2016). Muscle dysfunction in type 2 diabetes: a major threat to patient’s mobility and independence. *Acta Diabetologica*.

[43] Haider S., Ahmed S., Tabassum S., Memon Z., Ikram M., Haleem D. J. (2013). Streptozotocin-induced insulin deficiency leads to development of behavioral deficits in rats. *Acta Neurologica Belgica*.

[44] Kravitz A. V., Kreitzer A. C. (2012). Striatal mechanisms underlying movement, reinforcement, and punishment. *Physiology*.

[45] Durieux P. F., Bearzatto B., Guiducci S. (2009). D2R striatopallidal neurons inhibit both locomotor and drug reward processes. *Nature Neuroscience*.

